# The Importance of Species Traits for Species Distribution on Oceanic Islands

**DOI:** 10.1371/journal.pone.0101046

**Published:** 2014-07-08

**Authors:** Kristýna Vazačová, Zuzana Münzbergová

**Affiliations:** 1 Department of Botany, Faculty of Science, Charles University, Prague, Czech Republic; 2 Institute of Botany, Academy of Sciences of the Czech Republic, Průhonice, Czech Republic; INRA - University of Bordeaux, France

## Abstract

Understanding species' ability to colonize new habitats is a key knowledge allowing us to predict species' survival in the changing landscapes. However, most studies exploring this topic observe distribution of species in landscapes which are under strong human influence being fragmented only recently and ignore the fact that the species distribution in these landscapes is far from equilibrium. Oceanic islands seem more appropriate systems for studying the relationship between species traits and its distribution as they are fragmented without human contribution and as they remained unchanged for a long evolutionary time. In our study we compared the values of dispersal as well as persistence traits among 18 species pairs from the Canary Islands differing in their distribution within the archipelago. The data were analyzed both with and without phylogenetic correction. The results demonstrate that no dispersal trait alone can explain the distribution of the species in the system. They, however, also suggest that species with better dispersal compared to their close relatives are better colonizers. Similarly, abundance of species in the archipelago seems to be an important predictor of species colonization ability only when comparing closely related species. This implies that analyses including phylogenetic correction may provide different insights than analyses without such a correction and both types of analyses should be combined to understand the importance of various plant traits for species colonization ability.

## Introduction

Species ability to disperse and colonize new habitats is a key prerequisite for their response to ongoing landscape and climate changes [1], [2]. Understanding, which are the main traits responsible for this ability, is thus fundamental for prediction of future fates of different species [3], [4]. Many recent studies are attempting to understand the importance of species traits for species ability to colonize habitats of different size and isolation (e.g. [5], [6]). Most of these studies are done in various fragmented landscapes, predominantly in grasslands and forests. Often these studies demonstrate that species distribution is not only determined by current landscape structure, but is largely a result of landscape structure in the past (e.g. [7], [8]).

Strong species response to past landscape structure can be attributed to slow growth dynamics of many perennial species in combination with relatively fast changes in the current landscapes [9], [10]. Due to dispersal limitation [11], [12] and extinction debt [13], [14] the distribution of these species may reflect historical habitat configuration. Species distribution in the landscape may then not reflect species long-term ability to successfully colonize habitats and to survive there. Thus the traits driving species distribution on young habitat fragments in a changing landscape can be different from those in the landscapes fragmented for longer evolutionary time [15], [16].

Due to intensive human activity all over the world, it is rather difficult to identify fragmented habitats which remained unchanged for a long time period, on which we could study species ability to colonize new habitats on long time scales. Oceanic islands seem to be suitable candidates of such systems [17], [18]. In contrast to continental landscape, oceanic fragments are not a result of human activity and remained almost unchanged in size and number since their origin. Thus the islands are generally thought to be more stable in time as they are fragmented and isolated for much longer time periods. For these reasons they are suitable systems for studying the importance of dispersal traits for species occurrence on isolated patches. Similarly to the studies on habitat fragments on the mainland (e.g. [19], [20]), we can predict that species occurring on the youngest and the most isolated islands will have higher dispersal ability than species present on older and more connected islands.

In this study, we analyzed species traits determining distribution of selected native species on the Canary Islands. The Canary archipelago is a suitable model system as it consists of islands differing in their age, size and isolation as well as in species composition. Specifically, we attempted to understand the determinants of species presence on the newest, smallest and most isolated island (El Hierro).

Because closely related species often share a wide range of biological traits, distribution of a species may be related to the traits under study or to other traits correlated with these traits that are characteristic for the whole clade to which the species belongs [21], [22]. Comparison of results of analyses with and without phylogenetic correction can help in distinguishing between the traits that are really responsible for a pattern and traits correlated with these within larger species groups. The necessity of phylogenetic correction is a highly debated issue (e.g.[21], [22], [23]) and it has been suggested that the phylogenetic and ecological explanations for species distribution in a landscape are not mutually exclusive (see also [24]). Separating the phylogenetic and ecological explanations for species distribution is thus difficult. It is, however, generally recognized that both of these types of analyzes should be considered when trying to explain the effect of species traits on species distribution (e.g. [25]).

To consider species phylogeny in this study, we compared dispersal values of 18 pairs of closely related species differing in their presence on El Hierro. In addition, we used the same species to test the relationship between species traits and number of occupied islands in the archipelago. For each species, we assessed the dispersal ability by all possible dispersal vectors acting on islands, i.e. wind, water and animals (anemo-, hydro-, exo- and endozoochory). We also used published sources complemented with our field experience to identify the most likely dispersal mode for each species pair.

Even though nowadays some parts of the islands are quiet heavily inhabited, we suppose that the main dispersal events happened before human's strong influence. Also none of the studied species is purely ruderal. All the species occur in some (semi)-natural habitats such as laurel forests and canary pine woodlands. Such communities obviously suffer from human destructive activities being fragmented and reduced in area, but species extinctions on single islands occur only rarely and were not reported for any of our model species [26].

Although dispersal ability is widely considered as a major determinant of species distribution on islands due to their isolation, other traits, especially those related to species persistence on habitats should not be overlooked as was shown in studies e.g. by Maurer et al. [27] and Saar et al. [8]. For this reason we also tested traits related to species survival and persistence on the islands (i.e. species longevity and woodiness) and traits characterizing species distribution serving as a proxy for amount of seeds available (number of vegetation zones and number of islands occupied by a species). As a number of occupied islands itself can be a function of plant traits, we also explored the life history traits associated with number of occupied islands.

Specifically, we asked the following questions: 1) Which life history traits explain species presence on El Hierro? 2) Which life history traits explain the number of islands occupied by the species? 3) How do the conclusions change when applying phylogenetic correction?

We predict that species occurring on El Hierro will have better dispersal ability and will occupy more islands than species not occurring there. We also expect that species occupying more islands will be more likely r-strategists possessing traits, which enable rapid colonization of free space on islands (i.e. non-woody annuals occupying more vegetation zones).

## Materials and Methods

### Ethic statement

To test exozoochorous dispersal, we used a pigeon of the King breed, purchased from a local breeder. To minimize subjection to stress during the experiment, the animal was caged in its home aviary (2×1.5×1 m) and had free access to commercial diet and water. The bird was not subjected to any invasive intervention which could cause him suffering. As he was tamed since his youth, his manipulation during seed incorporation into feathers did not cause him extreme stress. The manipulation with pigeon was approved by Ministry of Education, youth and sport of the Czech Republic (permission no. 24773/2008-10001) and complied with the relevant legislation of the Czech Republic (article 11, regulation no. 207/2004).

### Study site

**Figure 1:**
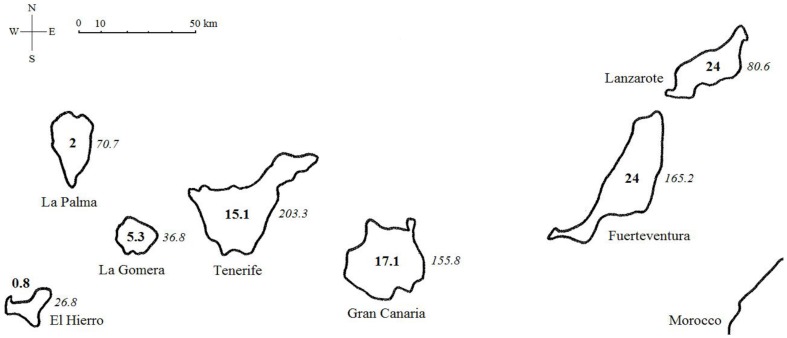
The Canary archipelago. Numbers in bold are island ages (in million years), numbers in italics are island areas (in hectares).

The Canary Islands are part of the Macaronesian archipelago situated between 27°45′ and 29°2′N and between 18°00′ and 13°37′W. They consist of 7 main volcanic islands differing in age and size (Figure 1). The age of the islands decreases with increasing distance from the closest mainland (Africa) and from east to west; the easternmost islands are the oldest, while the westernmost are the youngest. Vegetation composition and habitat diversity on islands is highly influenced by altitudinal gradients in combination with predominant north-eastern trade winds [28]. The oldest and most eroded islands Lanzarote and Fuerteventura lack forests, other, steeper and roughed islands (Gran Canaria, Tenerife, La Palma, La Gomera, El Hierro) are covered by thermo-sclerophyllous woodlands, evergreen laurel forests and canary pine woodlands. The highest parts of Tenerife and La Palma host meso-oromediterranean summit broom scrubs [29].

### Species selection

We selected 36 species belonging to 22 genera and 15 families, all native to the Canary Islands [30]. The species were grouped into pairs (Table 1). The species within the pair usually belong to the same genera. In three pairs, the two species in the pair represented closely related genera from the same family. Within each pair, the species differed in occurrence on El Hierro, on the youngest Canary Island, but they both were present on the adjacent islands (at least on Tenerife and La Palma or Tenerife and La Gomera). We chose Tenerife as it is considered as a centre of biodiversity of the area and thus can play a key role as a source for species dispersal to the westernmost islands ([31], but see [32]). La Gomera and La Palma were chosen because of their relative proximity to El Hierro and due to their similar size. All the three islands are also similar to El Hierro in the main vegetation zones including *Euphorbia* scrubs, thermo-sclerophyllous woodlands, evergreen laurel forests and canary pine woodlands. Due to these similarities we can suppose that species present on Tenerife and La Palma or Tenerife and La Gomera and not on El Hierro are those which have not been able to reach El Hierro due to dispersal limitation and not due to ecological barriers related to the absence of habitat [31].

**Table 1:** List of 18 species pairs used in the study (the first mentioned is species absent from El Hierro).

Species name^1^	Family	Analysed propagule	Most likely dispersal mode
*Aeonium sedifolium* (Webb *ex* Bolle) Pit. & Proust	Crassulaceae	Seed	ANEMO
*Aeonium spathulatum* (Hornem.) Praeger			
*Carex perraudieriana* Gay *ex* Bornm.	Cyperaceae	Seed	ANEMO [82]
*Carex canariensis* Kük.		(with utricle)	
*Cistus symphytifolius* Lam.	Cistaceae	Seed	ENDO [83]
*Cistus monspeliensis* L.			
*Euphorbia segetalis* L.	Euphorbiaceae	Seed	HYDRO [71] *
*Euphorbia lamarckii* Sweet			ENDO [72]
*Hypericum glandulosum* Aiton	Hypericaceae	Seed	ANEMO [84]
*Hypericum grandifolium* Choisy			
*Limonium imbricatum* (Webb *ex* Girard) C.F.Hubb.	Plumbaginaceae	Seed	EXO
*Limonium pectinatum* (Aiton) Kuntze		(with corolla)	
*Plantago ovata* Forssk.	Plantaginaceae	Seed	EXO [85]
*Plantago lagopus* L.			
*Polycarpaea aristata* (Aiton) DC.	Caryophyllaceae	Seed	ANEMO
*Polycarpaea nivea* (Aiton) Webb			
*Reichardia tingitana* (L.) Roth	Asteraceae	Achene	ANEMO [82]
*Reichardia ligulata* (Vent.) G. Kunkel & Sunding		(with pappus)	
*Reseda scoparia* Brouss. Ex Willd.	Resedaceae	Seed	ANEMO [82]
*Reseda luteola* L.			
*Salvia aegyptiaca* L.	Lamiaceae	Seed	EXO [86]
*Salvia canariensis* L.			
*Scrophularia glabrata* Aiton	Scrophulariaceae	Seed	ANEMO +BAL [79]
*Scrophularia arguta* Aiton			
*Senecio leucanthemifolius* Poir.	Asteraceae	Achene	ANEMO [82]
*Senecio glaucus* L.		(with pappus)	
*Tolpis lagopoda* C.Sm. *in* Buch	Asteraceae	Achene	ANE [85]
*Tolpis barbata* (L.) Gaertn.		(with pappus)	
*Trifolium stellatum* L.	Fabaceae	Seed	EXO [87]
*Trifolium arvense* L.		(with calyx)	
*Emex spinosa* (L.) Campd.	Polygonaceae	Seed	HYD [88] (*Emex*), [72] (*Rumex*)
*Rumex bucephalophorus* L.		(with spines)	EXO [89] (*Emex*)*, [90] (*Rumex*)*
*Monanthes laxiflora* (DC.) Bolle	Crassulaceae	Seed	ANEMO
*Aichryson laxum* (Haw.) Bramwell			
*Descurainia millefolia* (Jacq.) Webb & Berthel.	Brassicaceae	Seed	EXO [87] (*Descurainia*)
*Arabis caucasica* Schltdl.			[91] (*Arabis*)

1 The species names according to Arechavaleta et al. [30].

*The dispersal mode used as the most likely dispersal mode in the analyses presented (the results do not change when using the other dispersal mode).

We are aware that species presence/absence on El Hierro could be potentially mediated also by human activities. However, this island is less inhabited than the other Canary Islands. While some of the selected genera may occur in ruderal habitats (e.g. the genus of *Reseda*, *Senecio*, *Trifolium*), all of these occur also in some (semi)-natural habitats such as laurel forests and canary pine woodlands and could thus be distributed on the islands prior to increased human activities. We thus suppose that the main dispersal events happened before human's strong influence.

Species selection was further limited to species for which sufficient seed samples could be obtained. For this reason we had to exclude all the previously considered species pairs having fleshy fruits.

### Diaspore collection

Diaspores (fruits or seeds representing the most probable dispersal units, see Table 1) for each species were collected in natural populations on the islands except for *Limonium* species. Diaspores of *Limonium* were obtained from the populations in the Botanical Garden “Jardín Canario Viera y Clavijo”, Gran Canaria. The garden populations originally come from the island populations.

The collection from protected areas was done in cooperation with the Botanical Garden “Jardín Canario Viera y Clavijo”, Gran Canaria which obtained appropriate permission for collecting seeds for scientific purposes. The permission was issued by Consejería de Medio Ambiente y Aguas, Islas Canarias. The permission for seed collection from unprotected areas was not required.

In the field we preferably sampled 3 populations per species. For each population, we aimed to collect diaspores from at least 8 individuals. Each population was then tested for dispersal abilities separately. Garden collection was considered as one population and we sampled seeds from 8 individuals in the garden. To have the same number of measurements for the species with seeds collected from the field and from the garden, we had 3 replicates for each dispersal experiment for diaspores collected in the garden.

We used 20 diaspores per species and population for experiments with anemochory, hydrochory and exozoochory and 30 diaspores for testing endozoochory, i.e. 60 and 90 diaspores, respectively. Such number was a compromise between a large amount of species tested and number of seeds used in the literature (c.f. [33]).

For testing other traits related to dispersal (i.e. seed mass and seed viability) we used simple seeds, not fruits. In dispersal modes, where we used fruits as dispersal units, but accounted also for seed viability (i.e. hydrochory and endozoochory), the number of all seeds extracted from the fruits was used as a baseline number of seeds.

Data on all traits used in the study are provided in Supplementary Information (Table S1 and S2).

### Traits related to dispersal

#### Anemochory

The ability of diaspores to disperse by wind was estimated as terminal velocity defined as the maximum rate of seed falling in still air [34]. It was measured as the flight time of a diaspore from predefined height (270 cm [35]). Mean dispersal distance D was expressed as:
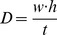
where w is the wind speed (being constant for all species), h is the average plant height and t is the terminal velocity. Values of average plant height were obtained from the literature [36], [37], [38], [39], [40]

We are aware that our dispersal model is simplified. Nevertheless, it has been successfully used in other studies to characterize mean dispersal distance of diaspores (e.g. [11], [6]) and is the easiest way to combine the three key variables affecting wind dispersal. We thus suggest that it is a useful proxy of potential wind dispersal distances for comparison among species.

In the analyses, we used both terminal velocity (m/s) and mean dispersal distance (m). In addition, we tested for the difference in plant height between species present on El Hierro and species absent from El Hierro to see to what extent are the differences in dispersal distance affected by differences in plant height.

#### Hydrochory

The potential of diaspores to disperse in salt water (buoyancy) was measured as the proportion of diasporess still floating after a defined time period. Diaspores were gently put into beakers filled with salt water having 3.7% salinity (i.e. average salinity of the Atlantic Ocean along the Canary Islands coast). The size of beakers was proportional to the size of diaspores. Sea waves were simulated by continual shaking in electric orbital shaker with frequency of 100 shakes per min. The number of diaspores floating on water surface was checked immediately after putting them into bins and then after 5 minutes of shaking, 1, 2, 6, 24 hours and 7 days of shaking [41]. The experiment was finished after 1 week of diaspores shaking as it is the minimal time a diaspore needs for reaching the Canary islands from mainland when taking into account average speed of water currents in the Atlantic Ocean (60–90 km per week [42]) and the distance between mainland and the closest island (Africa to Fuerteventura, 96 km).

At the end of the experiment, the number of floating and number of sunk diaspores was counted and the two groups of diaspores were then tested for seed viability.

In the analyses, we used the proportion of viable seeds which kept floating until the end of the experiment from the total number of viable seeds before the experiment.

The diaspore buoyancy was also expressed as T_50_, the number of minutes, after which 50 percent of diaspores was still floating. This parameter is commonly used in other studies assessing hydrochory [43], [41] however it does not take into account seed viability.

We also used the information on effect of salt water on viability of seeds expressed as the proportion of viable seeds after the experiment (both floating and sunk)/seed viability before the experiment. Viability of seeds was tested by dying the seeds with 0.1% solution of 2,3,5-triphenyl-2H-tetrazolium chloride [44]. In contrast to germination tests, it is not dependent on selection of the right conditions for germination for each individual species and it is thus in fact more reliable for between species comparisons.

#### Zoochory

Birds are the most important long-distance island dispersers transporting diaspores both externally and internally. The main bird dispersers acting on the Canary Islands are blackbirds (*Turdus merula*), robins (*Erithacus rubecula*), blackcaps (*Sylvia atricapilla* and *S. melanocarpa*, [45]), common ravens (*Corvus corax*, [46]), gulls (*Larus cachinnans*, [47]) and pigeons (*Columba livia*, *C. junoniae* and *C. bolli*).

#### Bird exozoochory (Epizoochory)

Bird exozoochory was tested as diaspore adhesion to bird feathers. As a model species we used a pigeon of the King breed, a utility breed with poor flight ability that is amenable to our experiments.

Although this species is clearly not native to Canary Islands, the functionality of its feathers for diaspore dispersal is readily comparable with native insular pigeon species.

As the seed coat of some species (e.g. *Plantago, Arabis*) contains mucilaginous substances which become sticky when wet, all the diaspores were moistened before the application into pigeon feathers. Moistened diaspores were gently incorporated into feathers on 4 different body parts (on bust, neck and back, under wing). After 1 hour of pigeon free movement in an aviary we checked the numbers of diaspores still attached to feathers. Taking into account the average flight speed of a trained pigeon (80 km/h [48]) and the shortest distance between mainland and the closest island (96 km), diaspores which remained attached to feathers after 1 hour are potentially able to get to the islands by this type of dispersal.

In the analyses we tested the proportion of diaspores which kept attached to feathers after 1 hour (we refer to this value as seed adhesion). This parameter lacks the effect of real bird flight as we do not take into account the air movement around feathers during the flight that can dry out diaspores and cause them to drop earlier than in our simulation. However some behavior of our pigeon during seed testing such as cleaning of feathers was similar to behavior of wild birds. Thus, we still think that our data are sufficient for the purpose to differentiate among diaspores with different ability to disperse by exozoochory.

#### Bird endozoochory

Bird endozoochory was tested by simulating diaspore gut passage through pigeon digestive tract. Plastic flasks filled up with diaspores were shaken with wet grit (small stones eaten by birds to enhance digestion, commercial mixture for pigeons) for 24 hours in electric orbital shaker (200 shakes per minute [49]). Then diaspores were separated from the grit, rinsed and immersed in 5 ml of 1 M H_2_SO_4_ (pH≈0.3 [50]) for 4 hours. Intact seeds were retrieved, counted and tested for viability. The proportion of number of viable seeds which survived the simulation to the number of seeds viability before the experiment was used in the analysis. Seed viability after simulation was tested as described above.

### Seed mass

Altogether 90 seeds per species were weighted. For this purpose, they were divided to groups by 10 to 30 seeds per group (10 seeds in the group for the largest and 30 for the smallest seeds, to get reasonable size estimates given by the precision of the balance, 0.0001 g). Seed mass is generally recognized as a rough proxy of seed dispersal ability and germination ability (e.g. [51], [6]). The same amount of seeds was used for viability testing of intact seeds.

### Most likely dispersal mode

For all species pairs the most likely dispersal mode was estimated from available literature (Table 1). Where such data were missing, we estimated the dispersal mode according to our experience with dispersal and diaspore morphology of the species.

### Traits related to persistence and distribution

Data on species longevity (short-lived vs. perennial), woodiness (woody vs. not woody) and the number of vegetation zones with species occurrence were gained from Bramwell and Bramwell [38] and Schönfelder and Schönfelder [39], [40].

Species distribution was expressed as a number of occupied islands, according to Arechavaleta et al. [30].

### Data analysis

To test the importance of life history traits for species presence on El Hierro, we used a generalized linear model with binomial distribution. Species category (present on El Hierro vs. absent from El Hierro) was used as dependent variable and species traits as independent variables. In this analysis the number of islands occupied by a species was counted excluding El Hierro as the effect of El Hierro is already included in the dependent variable.

The importance of traits for species distribution among islands was tested by log-normal regression. Number of islands occupied by a species was used as dependent variable and species traits as independent variables.

The analyses were also performed with phylogenetic correction. Because the exact phylogenetic relationships between the studied species are unknown, we used the simplest version of phylogenetic correction based on comparison of species within the pairs (e.g. [6]). The corrected trait values PC were calculated by applying the formula:
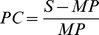
where S is the trait value of a single species (either present or absent from El Hierro) and MP is the mean of the trait value for each species pair. The phylogenetically corrected trait values were used in the tests as described above.

All the tests were done using two different approaches. First, we tested the effect of each trait separately. Afterwards, we combined all the traits in a single model and used forward step wise regression to select an optimal model.

To visualize the similarity between different species in their traits we used principal component analysis (PCA). The data on single species traits were treated as “species”, and data on each species represented “samples.” The analysis was centered and standardized by “species”; in this way all the traits were expressed in the same, relative, units.

Box plots were done in Statistica 7.0 [52], PCA was processed in CANOCO 4.5 [53]. All the other analyses were done in S-plus 6.2 Professional [54].

## Results

**Figure 2:**
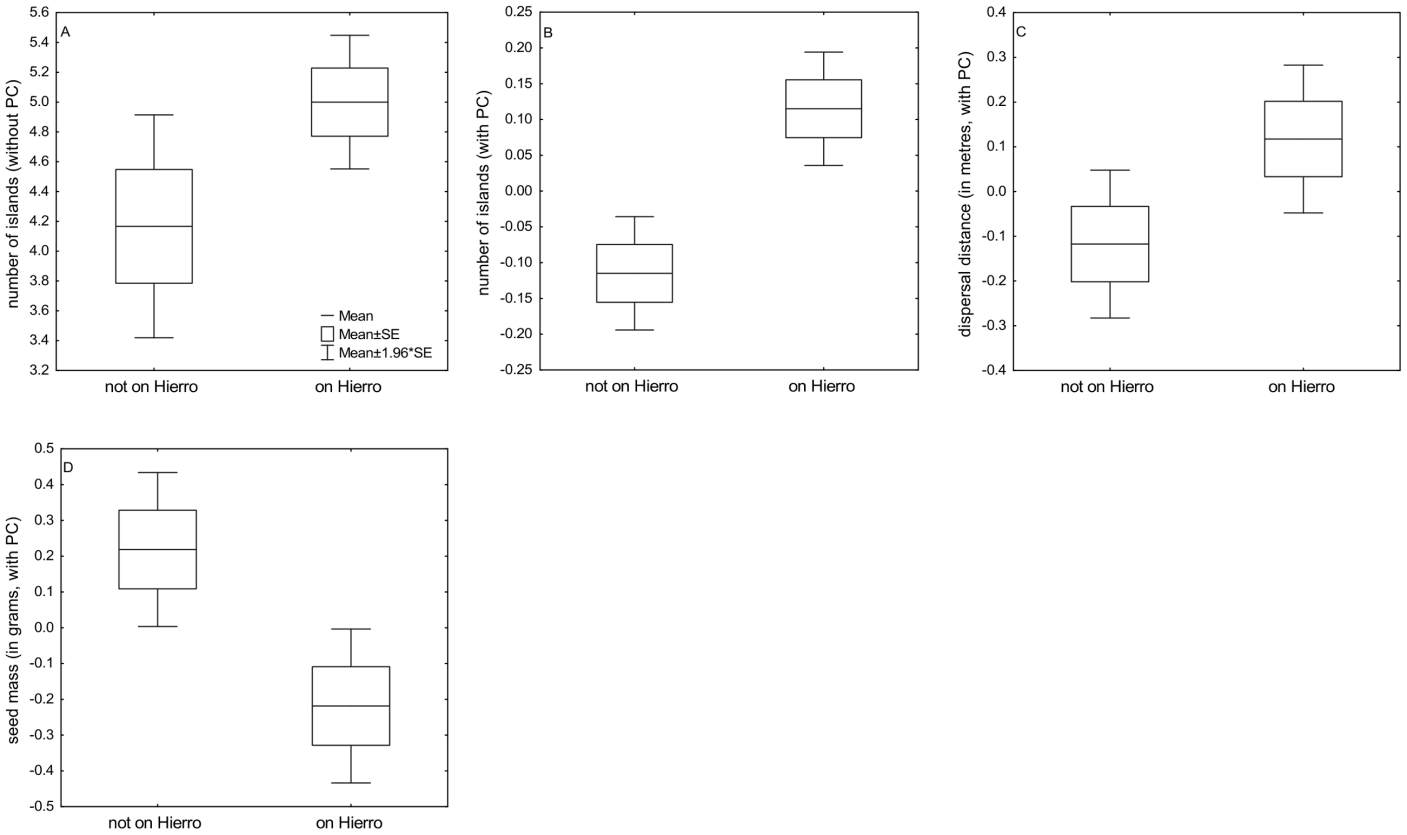
Box plots showing the differences between species present on El Hierro and species absent on El Hierro in number of islands occupied by a species without phylogenetic correction. (PC, A, p = 0.062) and with PC (B, p<0.001), dispersal distance with PC (C, p = 0.05) and seed mass with PC (D, p = 0.007).

Species present on El Hierro and species absent from El Hierro did not differ in any studied dispersal traits. There was, however, marginally significant effect on number of occupied islands (without El Hierro) (Table 2) with species present on El Hierro occupying more islands than species absent from El Hierro (Figure 2A). The results changed dramatically after incorporating phylogenetic correction into analyses. After phylogenetic correction, species presence on El Hierro was significantly influenced by dispersal distance, seed mass, species longevity and by the number of islands occupied by the species (Table 2). Species present on El Hierro dispersed further by wind, had smaller seeds, shorter life-span and occupied more islands than species absent from El Hierro (Figure 2B–D).

**Table 2:** Analysis of the relationship between the presence of species on El Hierro and the life history traits.

	Traits tested	Without PC				With PC			
		separately		By stepwise		separately		By stepwise	
		P	Dev	P	Dev	P	Dev	P	Dev
**Dispersal mode**									
ANEMOCHORY	terminal velocity	0.728	0.121			0.326	0.967		
	dispersal distance	0.983	<0.001			0.050	3.847	0.082	3.026
HYDROCHORY	buoyancy	0.927	0.009			0.553	0.353		
	seed survival in salt water	0.624	0.241			0.287	1.135		
	T50	0.686	0.164			0.384	0.759		
EXOZOOCHORY	seed adhesion	0.910	0.013			0.498	0.458		
ENDOZOOCHORY	seed viability after simulation	0.976	0.001			0.474	0.513		
MOST LIKELY DISPERSAL MODE		0.752	0.100			0.654	0.201		
**Other traits**	seed mass	0.317	1.000			0.007	7.338	0.021	5.340
	seed viability	0.990	<0.001			0.849	0.036		
	plant height	0.540	0.375			0.108	2.585		
**Persistence traits**	longevity	0.154	2.029			0.001	11.089	0.132	2.263
	woodiness	0.315	1.008			0.103	2.657		
**Distribution**	no. of vegetation zones	0.331	0.945			0.177	1.824		
	no. of islands	0.062	3.484	0.062	3.484	<0.001	15.14	0.002	9.487

The results are presented with and without phylogenetic correction (PC), Dev indicates deviance explained. DF Error = 35.

Number of islands occupied by a species was significantly influenced only by species longevity (Table 3). Species occupying more islands were more likely annuals than species occupying fewer islands. This trend remained the same even after phylogenetic correction. All the significant variables also remained in the model after stepwise regression showing that the traits are largely independent of each other (Tables 2 and 3).

**Table 3:** Analysis of the relationship between the number of occupied islands by a species and the life history traits.

	Traits tested	Without PC				With PC			
		separately		By stepwise		separately		By stepwise	
		P	Dev	P	Dev	P	Dev	P	Dev
**Dispersal mode**									
ANEMOCHORY	terminal velocity	0.492	0.472			0.513	0.428		
	dispersal distance	0.967	0.002			0.781	0.077		
HYDROCHORY	buoyancy	0.501	0.453			0.382	0.763		
	seed survival in salt water	0.862	0.030			0.643	0.215		
	T50	0.312	1.021			0.985	<0.001		
EXOZOOCHORY	seed adhesion	0.765	0.089			0.464	0.535		
ENDOZOOCHORY	seed viability after simulation	0.908	0.013			0.967	0.002		
MOST LIKELY DISPERSAL MODE		0.575	0.315			0.979	<0.001		
**Other traits**	seed mass	0.594	0.284			0.280	1.168		
	seed viability	0.465	0.534			0.425	0.637		
	plant height	0.518	0.418			0.995	<0.001		
**Persistence traits**	longevity	0.006	7.501	0.006	7.501	0.055	3.693	0.055	3.693
	woodiness	0.038	4.316			0.186	1.75		
**Distribution**	no. of vegetation zones	0.194	1.687			0.346	0.886		

The results are presented with and without phylogenetic correction (PC), Dev indicates deviance explained. DF Error  = 35.

**Figure 3:**
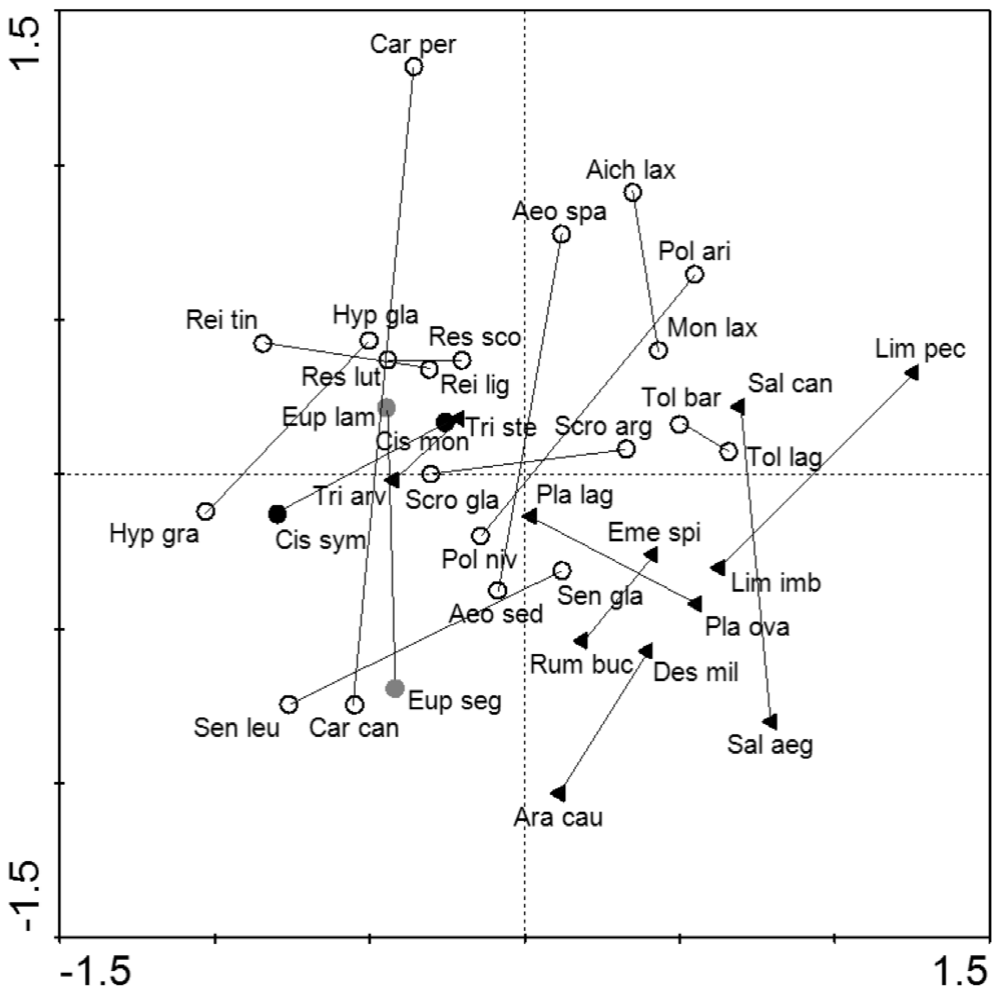
Relationship between individual species determined by principal component analysis (PCA) using trait data as dependent variables. The first axis explained 27.7% of variability, the second axis explained 26.1%. Different symbols indicate species most likely dispersal modes (according to literature): species with solid black circles are most likely dispersed by endozoochory, species with solid grey circles are dispersed by hydrochory, species with opened symbols are dispersed by anemochory and species with solid black triangles are dispersed by exozoochory. Species pairs are connected by lines.

Principal component analysis of dispersal traits showed that species within a pair are rather dissimilar in their traits (Figure 3). As seen in Figure 3, species are partly grouped according to the most likely dispersal mode.

## Discussion

The results of the study indicated that species presence on El Hierro, the smallest, youngest and the most remote island is influenced by both dispersal and persistence traits as well as by the number of other Canary Islands occupied by a species. This result was, however, found only after applying phylogenetic corrections. This suggests that the advantage of these traits is relative, and the traits thus play a role only after accounting for other possible differences between closely related species.

Contrasting results with and without phylogenetic correction were found previously also by e.g. Tremlová and Münzbergová [6] for dispersal traits, by Lanta et al. [55] for traits related to plant growth and by Stratton [56] for flower longevity pointing out the necessity for considering phylogenetic information in the analyses. The strong discrepancy between the two types of results is related to the stability of these traits within species phylogenies (e.g. [57], [58], [59]). The results obtained in this study should thus be interpreted not as the main effects of the given dispersal mode. In contrast, they e.g. suggest that within a given species group (sharing a wide range of biological traits) the species with relatively better dispersal are better colonizers.

Our expectation that species present on El Hierro disperse better than species absent from El Hierro holds only for wind dispersal mode. The importance of anemochory in dispersal among oceanic islands has been mentioned in classical islands studies [60], [61]. Regarding the Canary archipelago, seed transport from the eastern to the western islands (including El Hierro) can be mediated by northeasterly trade winds (which blew during arid Quaternary episodes [62]) as it was reported by e.g. Percy and Cronk [63] or Allan et al.[64]. However, when estimating dispersal distance using simply the data on terminal velocity, plant height and mean wind speed on islands (6.55 m/s [65]) and the nearest distance from El Hierro to neighboring island (La Gomera, 50 km), no species would be able to reach the island by wind. While such simple dispersal model is commonly used to approximate wind dispersal ability of species, such a model is rather simplified [66]. To estimate realistic dispersal distances of species we need to know also other parameters related to wind activity (mainly turbulence and updrafts) and island topography. Considering these types of data in the model is, however, beyond the scope of this study. Another indirect evidence for the importance of wind as an important dispersal mechanism on islands is that species present on El Hierro have smaller seeds (and thus more suitable for flying in the air) than species not present there. Generally, according to Lindborg et al. [25], species with smaller seeds are better dispersers, whereas those with large seeds are better recruiters and tend to have improved establishment in a wider range of habitats [67], [68] or when competing with neighbors (see [69]). However, the good competitive ability is not necessarily important for habitats on young volcanic islands arising de novo such as El Hierro. Additionally, the vegetation on El Hierro was repeatedly disturbed by volcanic activities causing extensive landslides further favoring good colonizers over good competitors.

No significant differences in other dispersal traits between species differing in the presence on El Hierro can signify that these species do not disperse by the tested dispersal modes in reality. For this reason we also tested the most likely dispersal mode, which was based on the selection of the most likely dispersal mode within species pair according to the literature. However, using the most-likely dispersal mode did not show significant differences between species present on El Hierro and absent from El Hierro. The use of such type of dispersal information from a variety of literary sources based on heterogeneous methodology for determining the most likely dispersal mode is questionable, but frequently practiced [70], [69]. As a result, the most likely dispersal mode differs according to different authors for some species (e.g. for *Euphorbia* hydrochory in Wald et al. 2005 [71] and endozoochory in Carlquist 1967 [72]). However, even after changing the most likely dispersal mode of some species there were no significant differences between species present and absent on El Hierro in their dispersal ability. Moreover, the only significant wind dispersal in our study was the most frequently chosen most likely dispersal mode. This suggests that the selection of the most likely dispersal mode is not so far from the reality.

According to our results, species present on El Hierro are distributed on more islands (excluding El Hierro) than species absent from El Hierro. This could be due to better wind dispersal ability of species on El Hierro. However, no dispersal trait significantly predicted number of islands occupied by the species. This suggests that better dispersal ability is not generally related to distribution on more islands as we could suppose. No relationship between dispersal and range size was shown e.g. by Kelly and Woodward [70], Goodwin et al. [73] and Lester et al. [74]. Lester et al. [74] assumed that dispersal may only influence species' geographical distributions at certain spatial scales or in particular habitats or environment and/or within certain taxonomic groups, depending on how the mechanisms by which dispersal and range size are related.

The reason why the number of occupied islands is a good predictor of species' presence on El Hierro could be that the number of occupied islands represents a measure of the amount of available sources (i.e. a proxy of number of plant populations or species abundance) for species' colonization (e.g. [75]). Indeed, to properly measure the amount of available sources we should also know the species local abundances and seed production. Obtaining good information on these two characteristics is, however, rather complicated and such data are not available. Alternatively, number of occupied islands could also be linked to niche width as species with wider distribution range tend to have wider niches and thus more likely occupy a novel habitat (Knappová unpubl.).

Species longevity was another trait influencing species presence on El Hierro.

Species on El Hierro were mainly short-lived (annuals and biennials) showing that short life span enabling rapid production of offspring can be an advantage for colonizing this westernmost island. Due to their ruderal strategy, short-lived species are usually able to grow on newly emerged or disturbed habitats indicating that the island vegetation is still developing. According to Kelly [76] and Kelly and Woodward [70] short-lived species are expected to have smaller ranges than perennials, which is in contrast to our results. We showed the opposite pattern; short-lived species have wider distribution among islands.

There are other possible traits such as seed bank longevity, seed production, pollination mode or detailed characteristics of species habitat requirements (e.g. in the form of indicator values) or species local abundance, which can influence species distribution as was shown e.g. by Pocock [23] and Gabrielová et al. [77]. These studies are mainly done on European species, where most of these data are available as a part of databases [78], [33]. No such complete data is, however, available for the whole flora of the Canary Islands.

### Possible limitations of the study

Despite the above arguments explaining limited role of dispersal traits in species distribution we cannot exclude the possibility that the importance of species dispersal is undervalued due to our species selection, especially by excluding species with fleshy fruits. We excluded species with fleshy fruits primarily for practical reasons as we were not able to collect sufficient number of fruits due to scarcity and the protection status of some of the potential species (e.g *Sambucus palmensis, Pleiomeris canariensis, Heberdenia excelsa*). However, as our species list involves mainly anemo- and exozoochorous species, addition of only few pairs of species with fleshy fruits would generate uneven distribution of dispersal modes resulting in few strong outliers. Such data could maybe lead to conclusion that dispersal is more important than we are suggesting based on the current results. On the other hand such a conclusion based on few outliers would not be very robust. We thus suggest that the limited species selection used in this study can also be viewed as an advantage as our study provides relatively robust conclusions for a wide range of anemo- and exozoochorous species.

Another possible critique of our study is that we are working with only 18 pairs of species. Species number was mainly limited due to the approach used to study dispersal, which was dependent on large number of seeds available for each species. Thanks to this approach, we were, however, able to obtain really detailed information on species dispersal by the main dispersal vectors acting among islands. In contrast, other dispersal studies dealing with more species are often based only on categorization of dispersal abilities inferred from the combination of seed visual observation and field experience [79], [80] or assessing dispersal by one dispersal mode only ([81]). Such approach enables to cover larger number of species, but species traits are only roughly assessed. As a result, the insights obtained in these studies are more general on one hand, but very rough on the other, not allowing to explore the importance of smaller differences in dispersal ability between different species. We suggest that the results obtained in our study are more likely to indicate possible long-term fates of species in fragmented systems within sets of species of similar growth forms dispersing in similar ways.

## Conclusions

The results demonstrated that the relationship between species distribution and species traits depends on the approach we use. Different results were obtained after incorporating phylogenetic relationship between species than when such correction was not used. Thus we suggest to combine both approaches when analyzing closely related species to understand the importance of various plant traits for species distribution.

## Supporting Information

Table S1 **Values of dispersal traits of 18 species pairs used in the study (the first mentioned is species absent from El Hierro).**
(DOC)

Table S2 **Values of persistence traits, traits related to distribution and other traits of 18 species pairs used in the study (the first mentioned is species absent from El Hierro).**
(DOC)

Checklist S1 **ARRIVE Checklist.**
(DOC)
