# Supplementary material for: The Importance of Species Traits for Species Distribution on Oceanic Islands
Source: PLoS One. 2014 Jul 8;9(7):e101046. doi: 10.1371/journal.pone.0101046 (PMC4086896; doi:10.1371/journal.pone.0101046)
Supplement: Table S1 — Values of dispersal traits of 18 species pairs used in the study (the first mentioned is species absent from El Hierro). (DOC) [file pone.0101046.s001.doc]

Table S1: Values of dispersal traits of 18 species pairs used in the study (the first mentioned is species absent from El Hierro)

| **Species** | ANEMOCHORY | | HYDROCHORY | | | EXOZOOCHORY | ENDOZOOCHORY | **most likely dispersal mode^6^** |
| --- | --- | --- | --- | --- | --- | --- | --- | --- |
|  | **terminal velocity (m/s)** | **dispersal distance (m)** | **buoyancy^1^** | **seed survival in salt water^2^** | **T50 (min)^3^** | **seed adhesion^4^** | **seed survival**  **after simulation^5^** |  |
| *Aeonium sedifolium* | 1.04 | 0.27 | 0.9 | 0.9 | 10080 | 0.2 | 0 | 0.27 |
| *Aeonium spathulatum* | 0.76 | 0.54 | 0.31 | 0.31 | 10080 | 0.05 | 0.1 | 0.54 |
| *Carex perraudieriana* | 1.35 | 1.69 | 0.35 | 0.35 | 10080 | 0.1 | 0 | 1.69 |
| *Carex canariensis* | 2.2 | 0.5 | 1 | 1 | 10080 | 0.32 | 0.43 | 0.5 |
| *Cistus symphytifolius* | 2.5 | 0.53 | 0.81 | 0.81 | 10080 | 0.15 | 0.78 | 0.78 |
| *Cistus monspeliensis* | 3.19 | 0.21 | 0.25 | 0.57 | 5281 | 0.13 | 0.85 | 0.85 |
| *Euphorbia segetalis* | 2.92 | 0.24 | 0.69 | 1 | 7920 | 0.28 | 0.63 | 0.69 |
| *Euphorbia lamarckii* | 3.28 | 0.36 | 0.41 | 0.78 | 6720 | 0.02 | 0.56 | 0.33 |
| *Hypericum glandulosum* | 0.89 | 1.31 | 0.77 | 0.77 | 10080 | 0.18 | 0 | 1.31 |
| *Hypericum grandifolium* | 1.12 | 0.96 | 1 | 1 | 10080 | 0.14 | 0.49 | 0.96 |
| *Limonium imbricatum* | 1.58 | 0.13 | 0.05 | 1 | 5760 | 0.3 | 0 | 0.3 |
| *Limonium pectinatum* | 1.2 | 0.17 | 0 | 0 | 5760 | 0.47 | 0 | 0.47 |
| *Plantago ovata* | 2.26 | 0.06 | 0.16 | 1 | 3195 | 0.3 | 0.04 | 0.3 |
| *Plantago lagopus* | 2.48 | 0.09 | 0.52 | 0.97 | 7620 | 0.05 | 0.01 | 0.05 |
| *Polycarpaea aristata* | 1.35 | 0.05 | 0.2 | 0.2 | 10080 | 0.05 | 0 | 0.05 |
| *Polycarpaea nivea* | 1.39 | 0.15 | 0.86 | 0.86 | 10080 | 0.08 | 0 | 0.15 |
| *Reichardia tingitana* | 0.24 | 0.85 | 0 | 1 | 900 | 0 | 0.91 | 0.85 |
| *Reichardia ligulata* | 0.49 | 0.87 | 0 | 0.97 | 410 | 0.15 | 0.52 | 0.87 |
| *Reseda scoparia* | 1.54 | 0.35 | 0.38 | 0.42 | 10080 | 0.12 | 0.62 | 0.35 |
| *Reseda luteola* | 1.82 | 0.49 | 0.56 | 0.6 | 10080 | 0.03 | 0.46 | 0.49 |
| *Salvia aegyptiaca* | 2.4 | 0.09 | 0.29 | 1 | 3382 | 0.53 | 0.01 | 0.53 |
| *Salvia canariensis* | 2.63 | 0.68 | 0.3 | 0.3 | 10080 | 0.47 | 0 | 0.47 |
| *Scrophularia glabrata* | 1.54 | 0.46 | 0.52 | 0.9 | 10080 | 0.12 | 0.32 | 0.46 |
| *Scrophularia arguta* | 1.55 | 0.25 | 0 | 1 | 45 | 0.1 | 0 | 0.25 |
| *Senecio leucanthemifolius* | 1.57 | 0.27 | 1 | 1 | 10080 | 0.2 | 0.57 | 0.27 |
| *Senecio glaucus* | 0.75 | 0.48 | 0 | 1 | 76 | 0.18 | 0 | 0.48 |
| *Tolpis lagopoda* | 1.7 | 0.21 | 0 | 0.73 | 63 | 0.25 | 0 | 0.21 |
| *Tolpis barbata* | 1.09 | 0.19 | 0 | 0.79 | 3330 | 0.15 | 0.02 | 0.19 |
| *Trifolium stellatum* | 1.74 | 0.09 | 0 | 0.66 | 900 | 0.1 | 0.97 | 0.1 |
| *Trifolium arvense* | 1.11 | 0.29 | 0.65 | 0.69 | 10080 | 0.12 | 0.59 | 0.12 |
| *Emex spinosa* | 3.02 | 0.11 | 0.16 | 1 | 90 | 0.2 | 0 | 0.2 |
| *Rumex bucephalophorus* | 1.72 | 0.15 | 0.25 | 0.8 | 5760 | 0.45 | 0.74 | 0.45 |
| *Monanthes laxiflora* | 0.47 | 0.17 | 0.35 | 0.35 | 10080 | 0.13 | 0 | 0.17 |
| *Aichryson laxum* | 0.5 | 0.66 | 0.12 | 0.18 | 10080 | 0.14 | 0.09 | 0.66 |
| *Descurainia millefolia* | 1.86 | 0.27 | 0.3 | 0.97 | 3382 | 0.45 | 0.27 | 0.45 |
| *Arabis caucasica* | 1.07 | 0.17 | 0.98 | 0.98 | 10080 | 0.5 | 0.21 | 0.5 |

^1^proportion of viable seeds which kept floating until the end of the experiment/seed viability before the experiment

^2^seed survival in salt water - the proportion of viable seeds after the experiment (both floating and sunk)/seed viability before the experiment.

^3^number of minutes, after which 50 percent of diaspores was still floating

^4^proportion of diaspores which kept attached to feathers after 1 hour

^5^proportion of viable seeds which survived the simulation/seed viability before the experiment

^6^values of the most likely dispersal mode estimated from literature
