# Supplementary material for: The Importance of Species Traits for Species Distribution on Oceanic Islands
Source: PLoS One. 2014 Jul 8;9(7):e101046. doi: 10.1371/journal.pone.0101046 (PMC4086896; doi:10.1371/journal.pone.0101046)
Supplement: Table S2 — Values of persistence traits, traits related to distribution and other traits of 18 species pairs used in the study (the first mentioned is species absent from El Hierro). (DOC) [file pone.0101046.s002.doc]

Table S2: Values of persistence traits, traits related to distribution and other traits of 18 species pairs used in the study (the first mentioned is species absent from El Hierro)

| **Species** | Other traits | | | Persistence traits | | Distribution | |
| --- | --- | --- | --- | --- | --- | --- | --- |
|  | **seed mass (g)** | **seed viability** | **plant height (m)** | **longevity** | **woodiness** | **no. of vegetation zones** | **no. of islands** |
| *Aeonium sedifolium* | 0.00003 | 0.56 | 0.28 | perennial | woody | 2 | 3 |
| *Aeonium spathulatum* | 0.00001 | 0.4 | 0.4 | perennial | woody | 1 | 5 |
| *Carex perraudieriana* | 0.00297 | 0.25 | 1 | perennial | non-woody | 1 | 4 |
| *Carex canariensis* | 0.00094 | 0.68 | 1.05 | perennial | non-woody | 1 | 5 |
| *Cistus symphytifolius* | 0.00052 | 0.99 | 1.3 | perennial | woody | 2 | 2 |
| *Cistus monspeliensis* | 0.00087 | 0.98 | 0.65 | perennial | woody | 1 | 5 |
| *Euphorbia segetalis* | 0.00234 | 0.88 | 0.7 | perennial | woody | 2 | 3 |
| *Euphorbia lamarckii* | 0.00857 | 0.89 | 1.15 | perennial | woody | 1 | 4 |
| *Hypericum glandulosum* | 0.00009 | 0.72 | 1.15 | perennial | woody | 1 | 5 |
| *Hypericum grandifolium* | 0.00004 | 0.9 | 1.05 | perennial | woody | 4 | 7 |
| *Limonium imbricatum* | 0.00121 | 1 | 0.2 | perennial | non-woody | 1 | 2 |
| *Limonium pectinatum* | 0.00013 | 0.61 | 0.2 | perennial | woody | 1 | 5 |
| *Plantago ovata* | 0.00311 | 0.97 | 0.13 | annual | non-woody | 1 | 6 |
| *Plantago lagopus* | 0.00061 | 0.87 | 0.2 | annual | non-woody | 2 | 7 |
| *Polycarpaea aristata* | 0.00003 | 0.57 | 0.07 | perennial | woody | 1 | 3 |
| *Polycarpaea nivea* | 0.00004 | 0.47 | 0.2 | perennial | woody | 1 | 6 |
| *Reichardia tingitana* | 0.00036 | 0.72 | 0.2 | perennial | non-woody | 3 | 6 |
| *Reichardia ligulata* | 0.00062 | 0.75 | 0.3 | perennial | woody | 3 | 6 |
| *Reseda scoparia* | 0.00016 | 0.77 | 0.5 | perennial | woody | 1 | 4 |
| *Reseda luteola* | 0.00024 | 0.8 | 0.85 | annual | non-woody | 2 | 6 |
| *Salvia aegyptiaca* | 0.00046 | 0.91 | 0.2 | perennial | woody | 1 | 5 |
| *Salvia canariensis* | 0.00119 | 0.5 | 1.75 | perennial | woody | 3 | 7 |
| *Scrophularia glabrata* | 0.00009 | 0.32 | 0.7 | perennial | woody | 2 | 2 |
| *Scrophularia arguta* | 0.00008 | 0.91 | 0.38 | annual | non-woody | 3 | 7 |
| *Senecio leucanthemifolius* | 0.0002 | 0.76 | 0.4 | annual | non-woody | 1 | 5 |
| *Senecio glaucus* | 0.00016 | 0.28 | 0.3 | annual | non-woody | 1 | 6 |
| *Tolpis lagopoda* | 0.00031 | 0.32 | 0.35 | perennial | woody | 1 | 3 |
| *Tolpis barbata* | 0.00007 | 0.72 | 0.2 | annual | non-woody | 2 | 7 |
| *Trifolium stellatum* | 0.00223 | 0.72 | 0.15 | annual | non-woody | 2 | 6 |
| *Trifolium arvense* | 0.00025 | 0.88 | 0.3 | annual | non-woody | 2 | 7 |
| *Emex spinosa* | 0.01872 | 0.94 | 0.33 | annual | non-woody | 1 | 7 |
| *Rumex bucephalophorus* | 0.0002 | 1 | 0.25 | annual | non-woody | 1 | 7 |
| *Monanthes laxiflora* | 0.00001 | 0.8 | 0.075 | perennial | woody | 3 | 6 |
| *Aichryson laxum* | 0.00001 | 0.56 | 0.3 | annual | non-woody | 3 | 6 |
| *Descurainia millefolia* | 0.00024 | 0.97 | 0.5 | perennial | woody | 3 | 3 |
| *Arabis caucasica* | 0.00015 | 0.97 | 0.18 | perennial | non-woody | 2 | 5 |
